# Huberine, a New Canthin-6-One Alkaloid from the Bark of *Picrolemma huberi*

**DOI:** 10.3390/molecules23040934

**Published:** 2018-04-17

**Authors:** Carlos López, Manuel Pastrana, Alexandra Ríos, Alvaro Cogollo, Adriana Pabón

**Affiliations:** 1 Instituto de Química, Universidad de Antioquia, Medellín 050010, Colombia; carlopez.udea@gmail.com; 2 Grupo Malaria, Facultad de Medicina, Universidad de Antioquia, Medellín 050010, Colombia; manuel.pastrana@udea.edu.co (M.P.); alexrioso@hotmail.com (A.R.); 3 Jardín Botánico Joaquín Antonio Uribe, Medellín 050010, Colombia; cogolloi@yahoo.com

**Keywords:** canthin-6-one, *Picrolemma huberi*, Simaroubaceae, antiplasmodial activity

## Abstract

A new alkaloid, Canthin-6-one, Huberine (**1**), together with three known compounds including 1-Hydroxy-canthin-6-one (**2**), Canthin-6-one (**3**) and stigma sterol (**4**), were isolated from the stem bark of *Picrolemma huberi*. The isolation was achieved by chromatographic techniques and the purification was performed on a C18 column using acetonitrile/water (90:10, *v*/*v*) with 0.1% formic acid as the mobile phase. The structural elucidation was performed via spectroscopic methods, notably 1D- and 2D-NMR, UV, IR, MS and HRMS. The antiplasmodial activity of the compounds was studied.

## 1. Introduction

**Figure 1:**
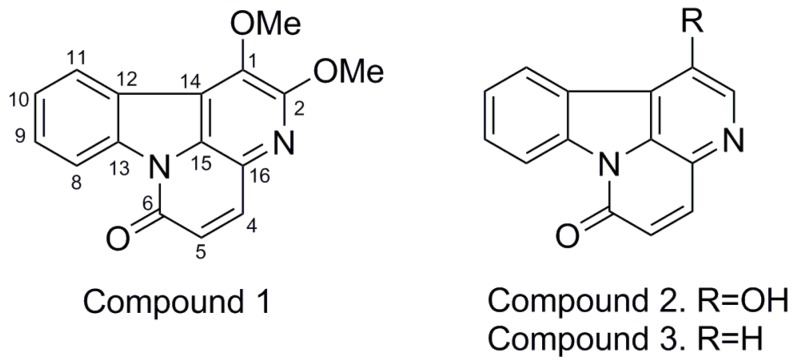
Canthin-6-one alkaloids isolated from *Picrolemma huberi* bark.

Plants of the family Simaroubaceae are widely used in traditional medicine for the treatment of diseases in different countries around the world. Species belonging to the genus *Picrolemma* (Simaroubaceae) have long been used in traditional medicine for their antitumoral and antimalarial properties [1]. Previous phytochemical investigations of *Picrolema huberi* revealed the presence of terpenoids and alkaloids. Among these compounds, quassinoids and canthin-6-ones are principal constituents of the *Picrolemma* species [2,3,4,5]. Canthin-6-ones are a subclass of tryptophan-derived β-carboline alkaloids, and are characterized by an additional ring, D, giving the 6H-Indolo(3,2,1-de) (1,5) naphthyridin backbone. A general biosynthetic pathway of canthin-6-one alkaloids starts from tryptophan as a precursor and produces tryptamine which condense with acetic or ketoglutarate units, giving rise to a series of β-carboline intermediates, each time more oxidized. Except canthin-6-one itself, which has a simple structure, all the canthin-6-one alkaloids isolated from plants are oxidized at any position from C-1 to C-11 of the skeleton to form hydroxy and/or methoxy derivatives [1,6,7,8,9]. Meanwhile, more than 60 canthin-6-one alkaloids have been isolated from natural sources, mainly plants from the Rutaceae and Simaroubaceae families [10]. A broad range of biological activities has been reported for canthin-6-ones, such as antitumor, antibacterial, antifungal, antiparasitic, antiviral, anti-inflammatory, antiproliferative, and aphrodisiacal properties [11]. In this paper, we report the results of an investigation of the stem barks of *Picrolemma huberi*. Three canthinone alkaloids have been isolated; one of which is new, Figure 1. All of these alkaloids are reported for the first time from the genus *Picrolemma*.

## 2. Results and Discussion

### 2.1. Identification of Isolated Compounds 

Identification of compound 1 from the *Picrolemma huberi* bark. Compound **1**, named Huberine, was isolated as an amorphous, pale-yellow solid. The HR LCMS spectrum of **1** showed a pseudomolecular ion peak, [M + H] at *m*/*z* 281.0926, corresponding to a molecular formula C_16_H_12_N_2_O_3_. A positive Dragendorff test was obtained, suggesting that **1** was an alkaloid. IR absorption bands of conjugated carbonyl group were observed at 1664 cm^−1^ and unsaturation 1630 and 1598 cm^−1^. The UV spectrum of **1** displayed absorption maxima at 227, 296, 356, and 376 nm, which were similar to those reported for canthin-6-one alkaloids [12]. The ^13^C-NMR and DEPT-NMR spectra for **1** indicated the presence of 16 carbon signals, including two methoxyls, six methines and eight quaternary carbon signals. All the proton and protonated carbon signals of **1** were assigned unambiguously by an 2D-HSQC (Heteronuclear Single-Quantum Correlation) experiment. In the ^1^H-NMR spectrum (Table 1), four mutually coupled aromatic protons at δ 8.67 (1H, d, *J* = 8.1 Hz, H-8), δ 7.66 (1H, t, *J* = 7.6 Hz, H-9), δ 7.51 (1H, t, *J* = 7.7 Hz, H-10) and δ 8.22 (1H, d, *J* = 7.6 Hz, H-11) were observed in the ^1^H-^1^H COSY spectrum, meaning that the ring A of compound **1** is not substituted.

Isolated vicinal doublets at δ 7.83 (1H, *J* = 9.6 Hz, H-4) and δ 6.82 (1H, *J* = 9.6 Hz, H-5) were characteristic of *cis*-coupled protons on the conjugated lactam ring of a canthin-6-one. A 2D-HMBC (Heteronuclear Multiple Bond Correlation) experiment further confirmed the structure of alkaloid **1**. In the spectrum, cross-peaks were found for H-4 (δ 7.83) with C-6 (δ 160.1) and C-15 (δ 130.3), H-5 (δ 6.82) with C-16 (δ 126.4), showing that these are the quaternary carbons that join the C and D rings.

The placement of the methoxy groups was deduced from the HMBC experiments. The methoxy signals showed clear HMBC correlations with the C at 141.1 and 154.9, assigned as C-1 and C-2. The assignment of quaternary carbons was established by HSQC and HMBC spectral data. Thus, the structure of Huberine **1** was established as 1,2-dimethoxycanthin-6-one, which is reported here for the first time. The new compound **1** showed no effective antiplasmodial activity at concentrations evaluated (from 100 μg/mL to 1.56 μg/mL) in *Plasmodium falciparum* strain FCR-3.

Identification of compound **2** and **3** from the *Picrolemma huberi* bark. The structures of the known compounds **2** and **3** were identified as 1 hydroxycanthin-6-one (**2**) [13,14] and canthin-6-one (**3**) [15], by spectroscopic data (^1^H-NMR,^13^C-NMR, 2D-NMR, and MS) and by comparison with published values. Although 1-hydroxycanthin-6-one (**3**) was isolated from the Simaroubaceae family, it has not been reported from *P. huberi*. Stigmasterol (**4**) was also isolated.

### 2.2. Antiplasmodial Activity In Vitro

The antiplasmodial activity of compounds **1**, **2** and **3** was evaluated in vitro against the multi-resistant strain of FCR-3 of *P. falciparum*. In the concentrations evaluated (from 100 μg/mL to 1.5 μg/mL), they did not show any activity. 

## 3. Materials and Methods

### 3.1. General Procedures

Spectra were recorded on the following instruments: UV: Shimadzu UV-250 UV-Visible spectrophotometer (Canby, OR, USA); IR: Perkin Elmer 1600 (Waltham, MA, USA); NMR: BRUKER 600 MHz (Silberstreifen, Rheinstetten, DE); HRMS to compound (**1**) were measured on a Xevo Q-Tof Waters^®^ spectrometer (Milford, MA, USA) and MS of compounds **2**, **3** and **4** were measured on a Nermag-Sidar R10-10C spectrometer (Argenteuil, FR) with a quadrupolar filter. All solvents, except those used for bulk extraction, were AR grade. Silica gel 60 F254 was used for column chromatography. Glass and aluminum-supported silica gel 60 F254 plates were used for preparative TLC. TLC spots were visualized under UV light (254 and 365 nm) after spraying with Dragendorff’s reagent for alkaloid detection.

### 3.2. Plant Material

The stem bark of *P. huberi* was collected from the village, La Guada Reserve, [coordinates: 06°52′006′′ N to 75°08′49.9′′ W, (1.662 msnm)], close to Amalfi, Antioquia, Colombia, in January 2017. A voucher specimen (Tobón Juan Pablo 2392) has been deposited in the Herbarium JAUM (Joaquín Antonio Uribe Botanic Garden of Medellín, Antioquia, Colombia).

### 3.3. Extraction and Isolation

Dried stem bark (1.5 kg) of *P. huberi* was defatted with n-hexane (3 L). The marc was extracted with MeOH-H_2_O (90:10) (6 L) by percolation for 72 h and the same material was re-extracted in the same manner. The extract was filtered and concentrated up to 1 L under reduced pressure, and then partitioned with EtOAc (2 L). The EtOAc layer was dried over anhydrous Na_2_SO_4_ and then concentrated under reduced pressure (0.5 L). This extract (30 g) was initially subjected to an acid-base extraction [11] to give CHCl_3_ alkaloid (2.0 g).

The crude alkaloid (2.0 g) was subjected to column chromatography over silica gel using CH_2_Cl_2_ gradually enriched with methanol as eluent to yield ten fractions (A−J).

Fraction A (102 mg) was chromatographed on a silica gel column and eluted with DCM-AcOEt (1:1) to give six subfractions, A1−A6. Fraction A1 (75 mg) was chromatographed by preparative TLC with CH_2_Cl_2_-MeOH (95:5) and further purified by preparative RP-HPLC using the mobile phase CH_3_CN/H_2_O (90:10), 0.1% formic acid to yield compound **1** (10 mg). Similar HPLC of fraction A2 (15.0 mg) yielded compound **2** (1.3 mg) eluting at 13.2 min, and HPLC of fraction A3 (38.0 mg) yielded compound **3** (1.0 mg) eluting at 9.5 min.

Antiplasmodial in vitro activity assay of each compound (from 100 μg/mL to 1.5 μg/mL) was evaluated in FCR3 strain. The diphosphate salt of chloroquine (≥98%, SIGMA C6628), evaluated in a range of 2000 nM to 2.3 nM, was used as a treatment control in each assay [16].

### 3.4. Spectral Data

*Huberine: 1,2-Dimethoxy-canthin-6-one* (**1**). Yellow amorphous powder; UV (MeOH, max, nm): 207, 266, 294, 354, 369. IR (KBr, n, cm^−1^): 1664, 1550, 1439, 1214, 1086, 753; ^1^H- and ^13^C-NMR data, see Table 1; MS: Waters LCT Premier (ESI-TOF) spectrometer at *m*/*z* 281.0926 [M + H]^+^; calcd. for C_16_H_12_N_2_O_3_, 281.0926.

*1-Hydroxy-canthin-6-one* (**2**). Yellow amorphous powder. UV (MeOH, nm): 210, 249, 256,288, 341, 415. IR (KBr, cm^−1^): 3276, 1567, 1600, 1629. ^1^H-NMR (600 MHz, MeOD-*d*_4_, δ in ppm, *J*), 8.60 (d, 1H, 8.4Hz), 8.35 (s, 1H), 8.27 (d, 1H, 7.7Hz), 8.02 (d, 1H, 9.7Hz), 7.69 (t, 1H, 8.4Hz), 7.58 (t, 1H, 8.0Hz), 6.80 (d, 1H, 9.7Hz). ^13^C-NMR (150 MHz, DMSO-*d*_6_, δ in ppm): 151.39 (C-1), 135.55 (C-2), 139.48 (C-4), 123.38 (C-5), 159.66 (C-6), 123.60 (C-8), 125.79 (C-9), 129.16 (C-10), 116.22 (C-11), 137.61 (C-12), 137.5 (C-13), 114.31(C-14), 133.32 (C-15), 128.11 (C-16). MS TOF ES^+^ spectrometer at *m*/*z* 237.0708 [M + H]^+^.

*Canthin-6-one* (**3**). Yellow amorphous powder. UV (MeOH, nm): 210, 249, 256,288, 341, 415. IR (KBr, cm^−1^): 3276, 1567, 1600, 1629. ^1^H-NMR (600 MHz, DMSO-*d*_6_, δ, ppm, *J*/Hz): 8.35 (1H, d, *J* = 4.8 Hz, H-1, 8.86 (1H, d, *J* = 4.8 Hz, H-2), 8.7 (1H, d, *J* = 9.7 Hz, H-4), 7.02 (1H, d, *J* = 9.7 Hz, H-5), 8.55 (1H, d, *J* = 8.1 Hz, H-8), 7.79 (1H, t, *J* = 7.6 Hz, H-9), 7.62 (1H, t, *J* = 7.6 Hz, H-10), 8.42 (1H, d, *J* = 7.8 Hz, H-11). MS TOF ES^+^ spectrometer at *m*/*z* 221.0715 [M + H]^+^. Supplementary material is available online.

## 4. Conclusions

Huberine, a new canthin-6-one alkaloid (**1**) and **3** known compounds (**2**, **3** and **4**) were isolated from the stem bark of *P. huberi*. The structure of the new compound (**1**) was elucidated by spectroscopic data Huberine (**1**); it was isolated from this plant for the first time. The isolates were screened for inhibitory activity against *Plasmodium falciparum* strains. Compounds **1**, and **2** showed no effective antiplasmodial activity.

## Figures and Tables

**Table 1:** ^1^H-NMR (600 MHz) and ^13^C-NMR (125 MHz) spectral data of compound **1** in CDCl_3_ (δ, in ppm, *J* in Hz).

Position	^1^H (ppm), *J* (Hz)	^13^C (ppm)	COSY Coupling	HMBC Coupling(^2,3^*J*)
1		141.3		OCH_3_
2		155.1		OCH_3_
4	7.83(d, 9.6)	138.2	H-5	H-5
5	6.82(d, 9.6)	125.6	H-4	
6		160.1		H-4
8	8.67(d, 8.1)	117.4	H-9	H-9
9	7.66 (t, 7.6)	130.4	H-8, H-10	H-11
10	7.51 (t, 7.7)	125.8	H-9, H-11	
11	8.22(d, 7.6)	124.9	H-10	H-9
12		123.5		H-10
13		140.1		H-9, H-11
14		130.3		
15		130.3		H-4
16		126.4		H-5
OCH_3_	4.15 (s)	54.7		
OCH_3_	4.20 (s)	61.3		

## Supplementary Materials

The following are available online. Figure S1: Huberine: 1,2-Dimethoxy-canthin-6-one (**1**). ^1^H-NMR (CDCl_3_, 600 MHz); Huberine: 1,2-Dimethoxy-canthin-6-one (**1**). ^13^C-NMR (CDCl_3_, 150 MHz); Huberine: 1,2-Dimethoxy-canthin-6-one (**1**). COSY H-H (CDCl_3_); Huberine: 1,2-Dimethoxy-canthin-6-one (**1**). HSQC (CDCl_3_); Huberine: 1,2-Dimethoxy-canthin-6-one (**1**). HMBC (CDCl_3_); Huberine: 1,2-Dimethoxy-canthin-6-one (**1**). HRMS; Huberine: 1,2-Dimethoxy-canthin-6-one (**1**). FT-IR, 1-Hydroxy-canthin-6-one (**2**). ^1^H-NMR (MeOD-d4, 600 MHz); 1-Hydroxy-canthin-6-one (**2**). ^13^C-NMR (DMSO-*d*_6_, 150 MHz); 1-Hydroxy-canthin-6-one (2). COSY H-H (MeOD-*d*_4_); 1-Hydroxy-canthin-6-one (**2**). HSQC (MeOD-*d*_4_); 1-Hydroxy-canthin-6-one (**2**). HRMS; Canthin-6-one (**3**). ^1^H-NMR (DMOS-*d*_6_, 600 MHz); Canthin-6-one (**3**). HRMS.
