# Supplementary material for: Huberine, a New Canthin-6-One Alkaloid from the Bark of Picrolemma huberi
Source: Molecules. 2018 Apr 17;23(4):934. doi: 10.3390/molecules23040934 (PMC6017910; doi:10.3390/molecules23040934)

SUPPLEMENTARY MATERIAL

Huberine, a New Canthin-6-one Alkaloid from the Bark of *Picrolemma huberi*.

López Carlos[1], Pastrana Manuel[2], Ríos Alexandra[2], Cogollo Alvaro[3], Pabón Adriana[2*]

1. Instituto de Química, Universidad de Antioquia, Medellín 050010, Colombia.
2. Grupo Malaria, Facultad de Medicina, Universidad de Antioquia, Medellín 050010, Colombia; E-Mail: apabon@udea.edu.co
3. Jardín Botánico Joaquín Antonio Uribe, Medellín 050010, Colombia.

* Author to whom correspondence should be addressed; E-Mail Adriana.pabonudea.edu.co

Huberine: 1,2-Dimethoxy-canthin-6-one (1). ^{1}H-NMR ($CDCl_3$, 600MHz).

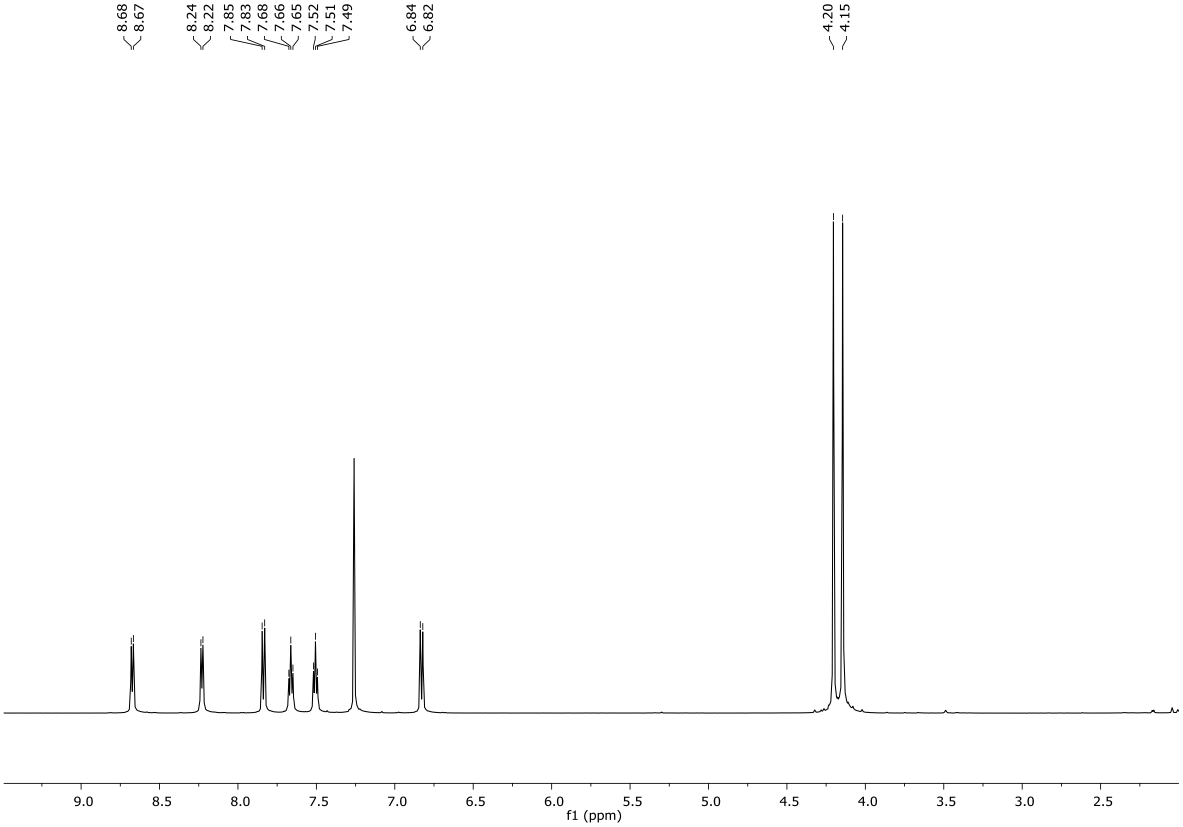

Huberine: 1,2-Dimethoxy-canthin-6-one (1). ^{13}C-NMR ($CDCl_3$, 150MHz).

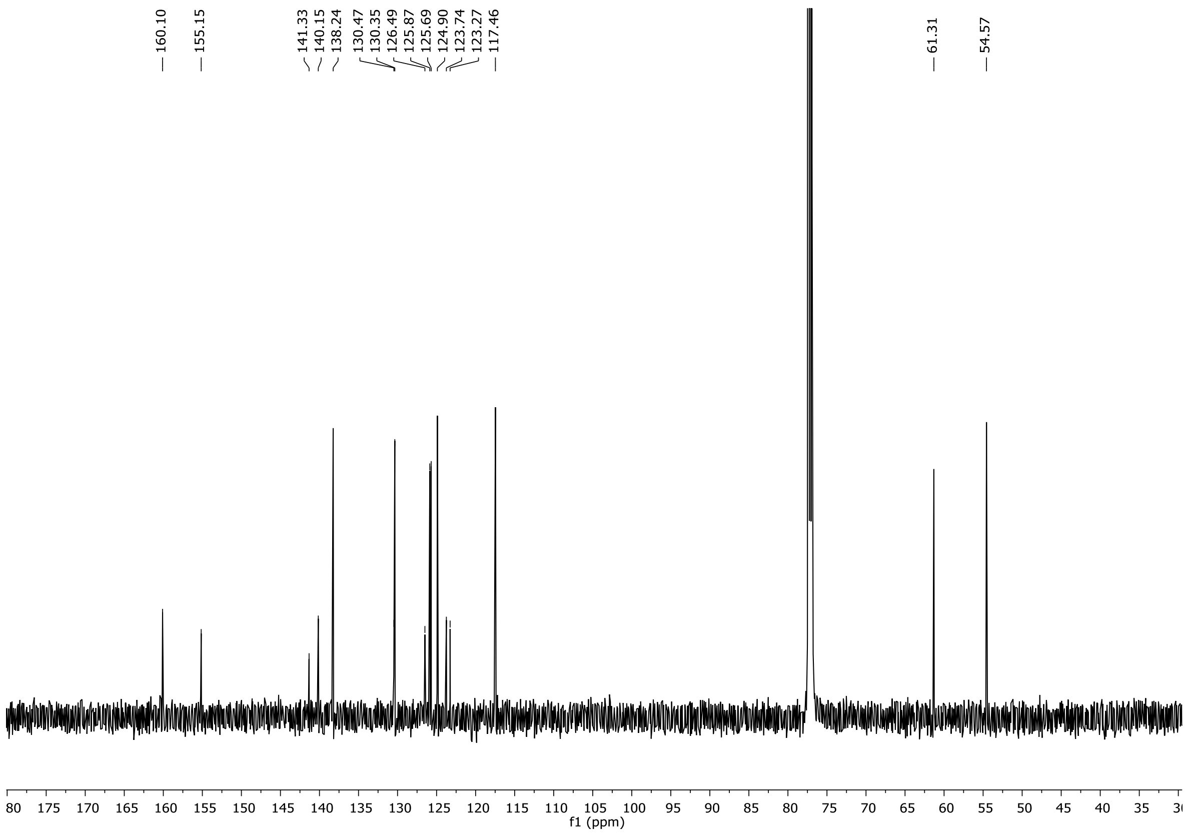

Huberine: 1,2-Dimethoxy-canthin-6-one (1). COSY H-H ($CDCl_3$)

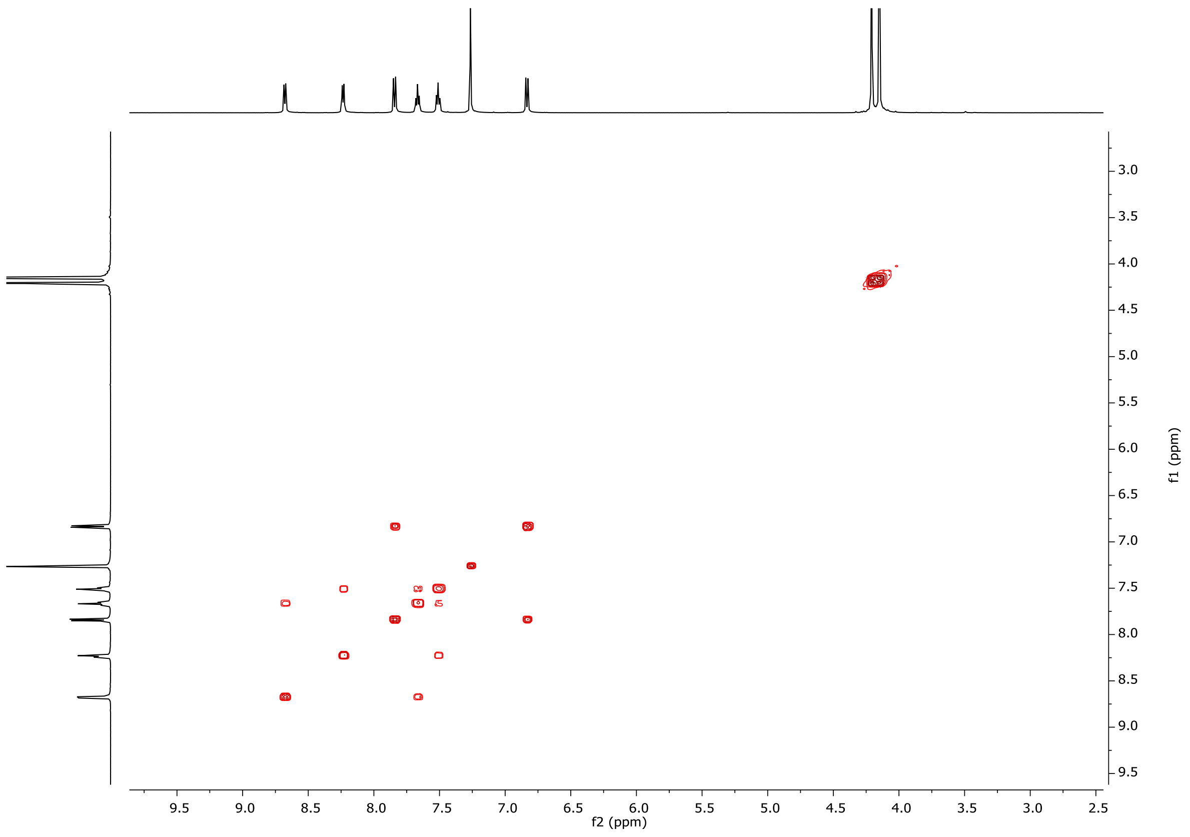

Huberine: 1,2-Dimethoxy-canthin-6-one (1). HSQC ($CDCl_3$)

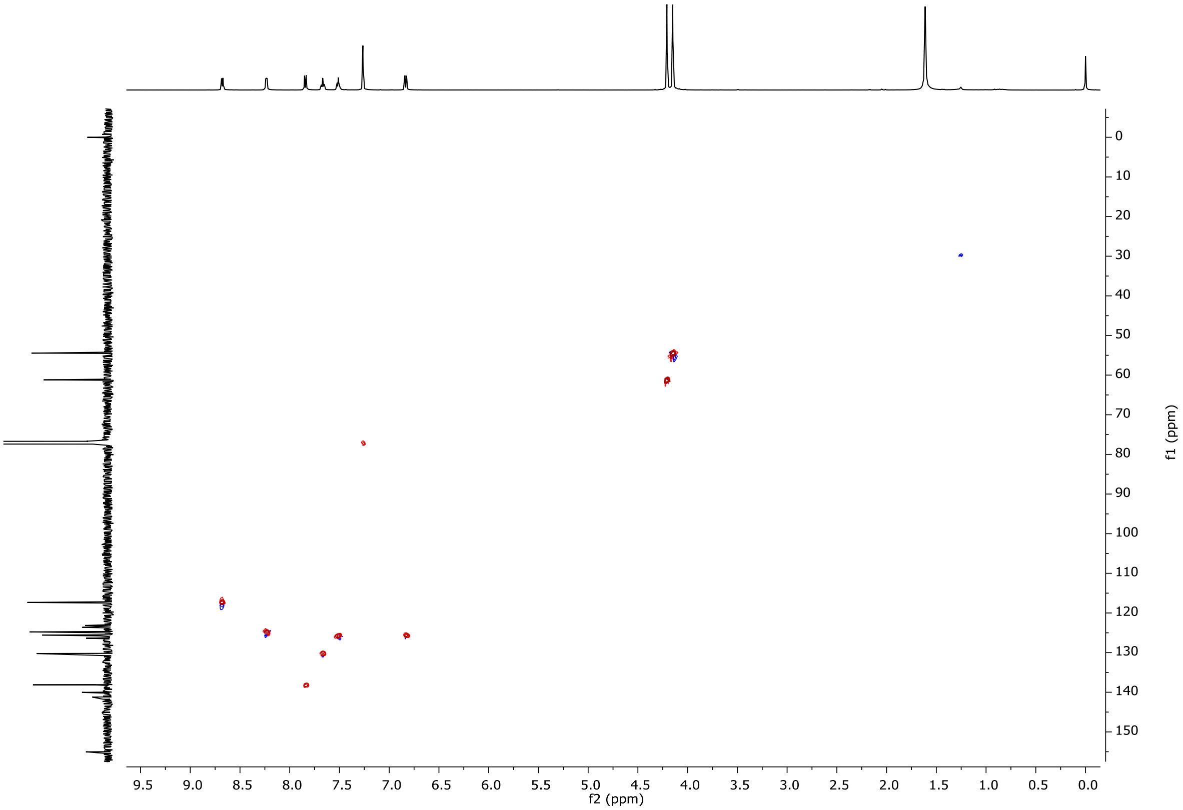

Huberine: 1,2-Dimethoxy-canthin-6-one (1). HMBC ($CDCl_3$)

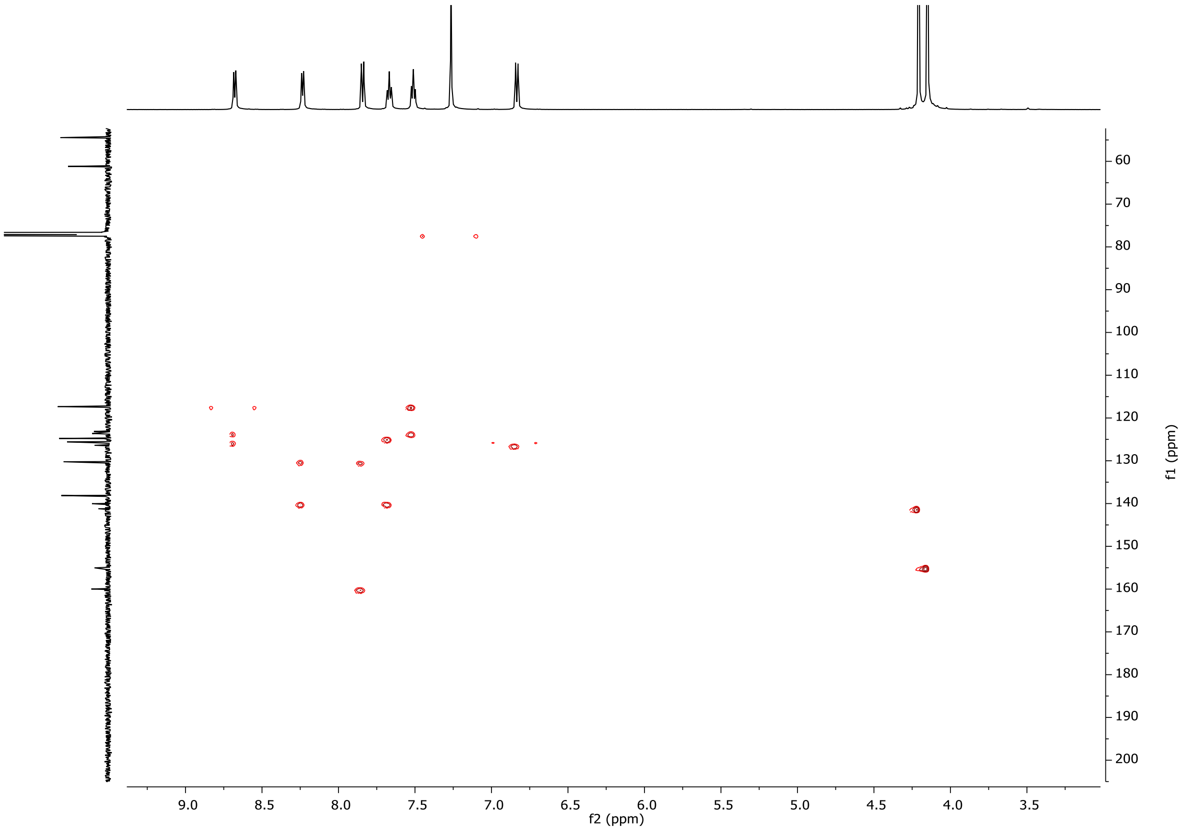

Huberine: 1,2-Dimethoxy-canthin-6-one (1). HRMS

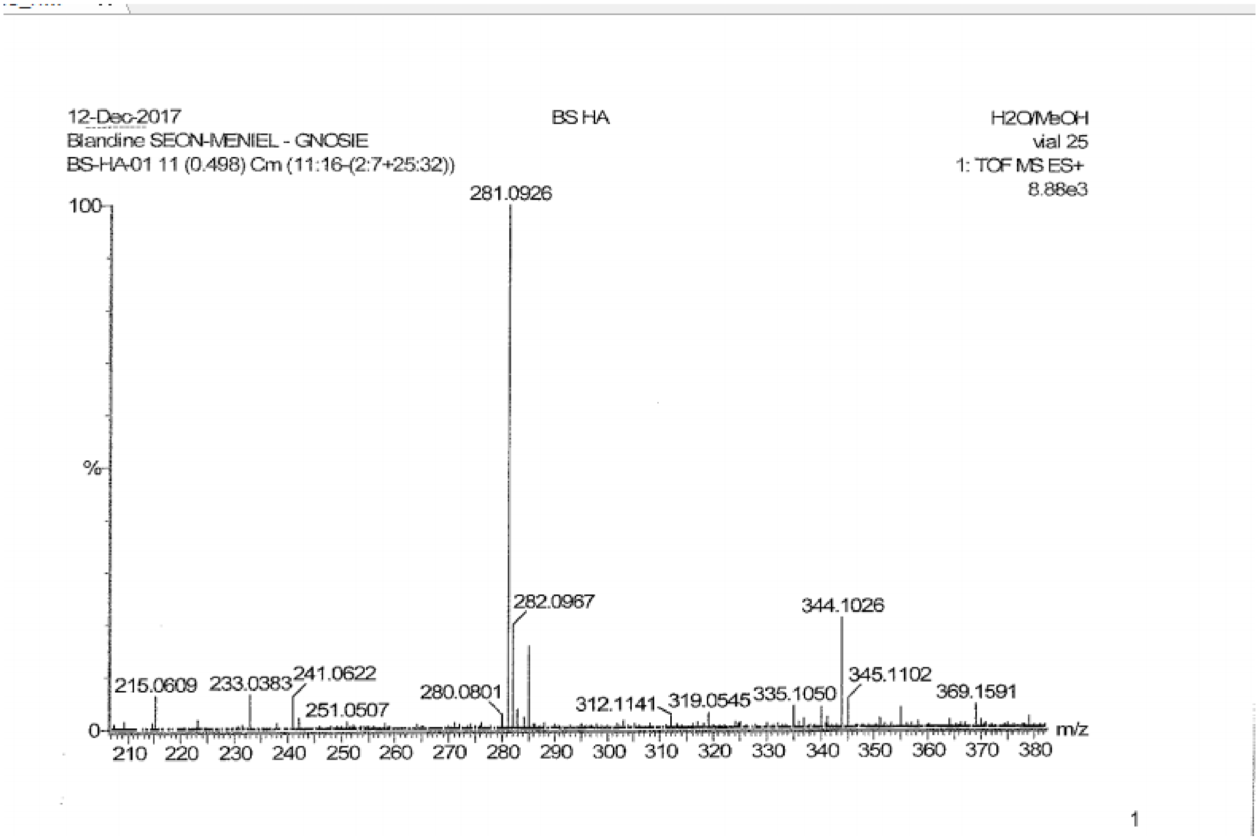

Huberine: 1,2-Dimethoxy-canthin-6-one (1). FT-IR

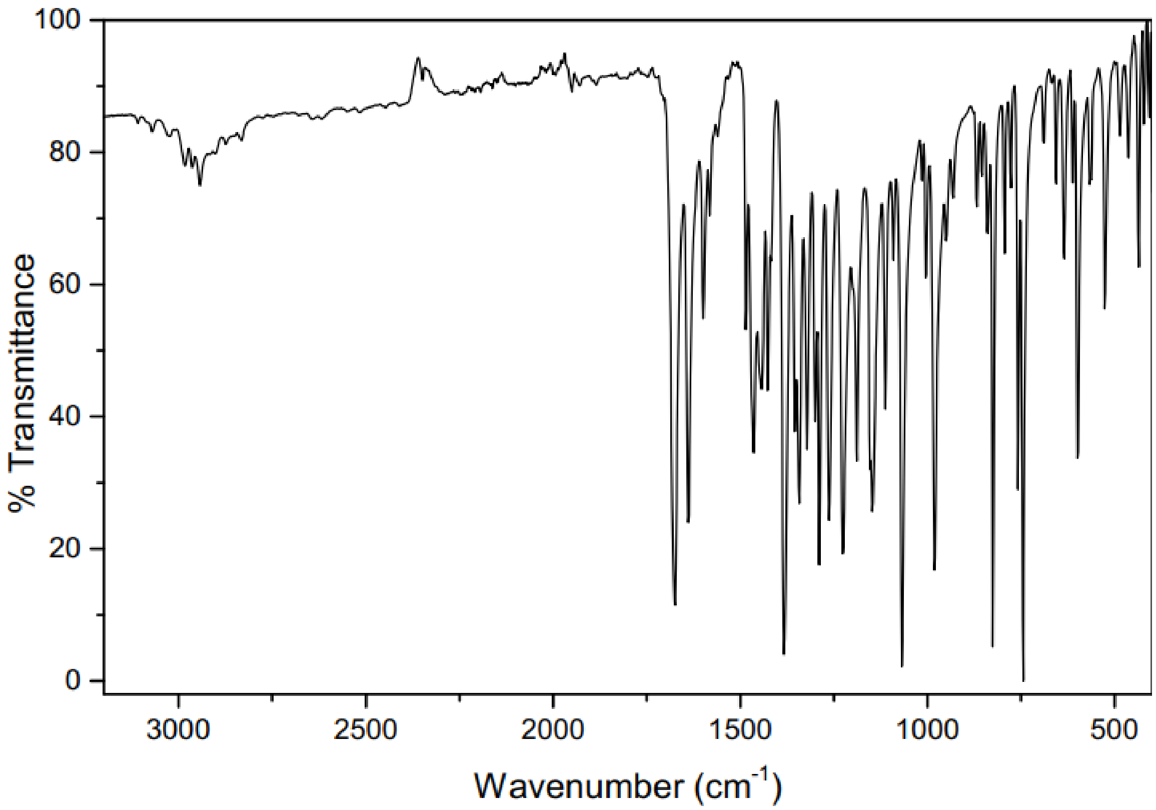

1-Hydroxy-canthin-6-one (2). ^{1}H-NMR (MeOD-d4, 600MHz).

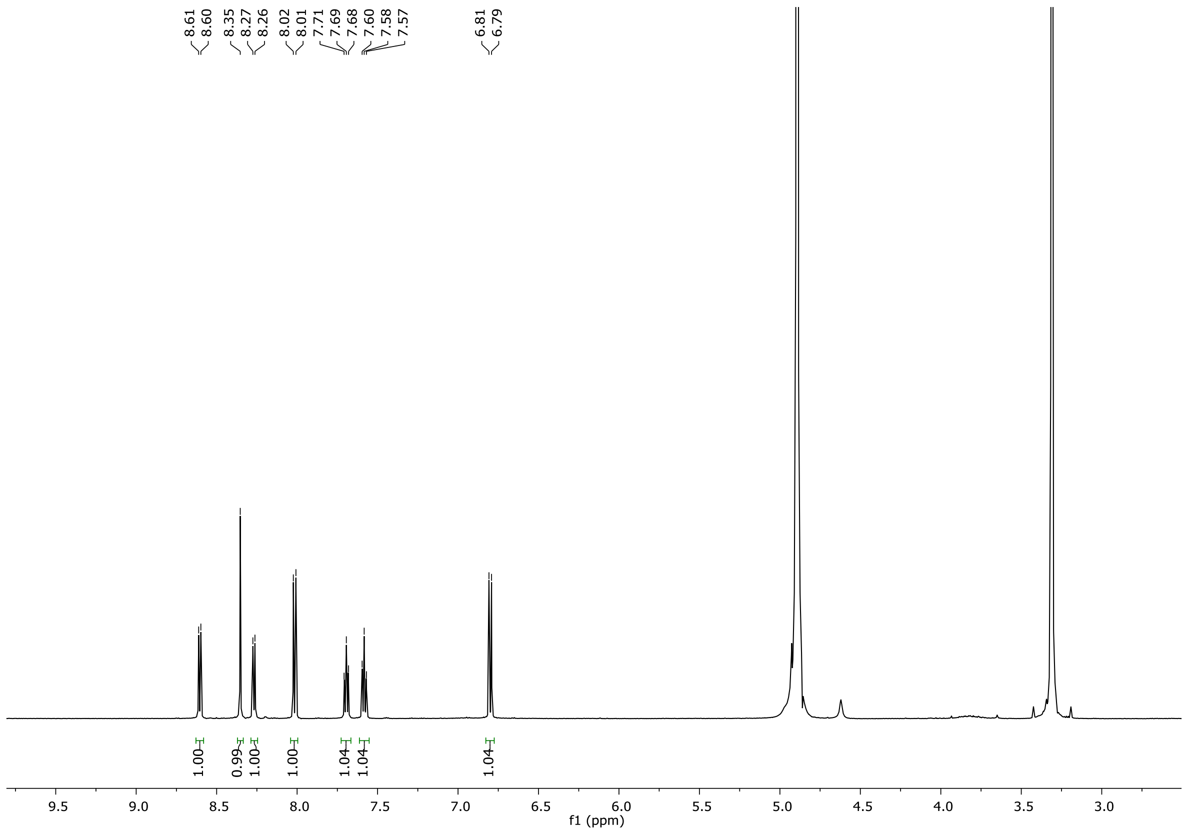

1-Hydroxy-canthin-6-one (2). ^{13}C-NMR (DMSO-d6, 150MHz).

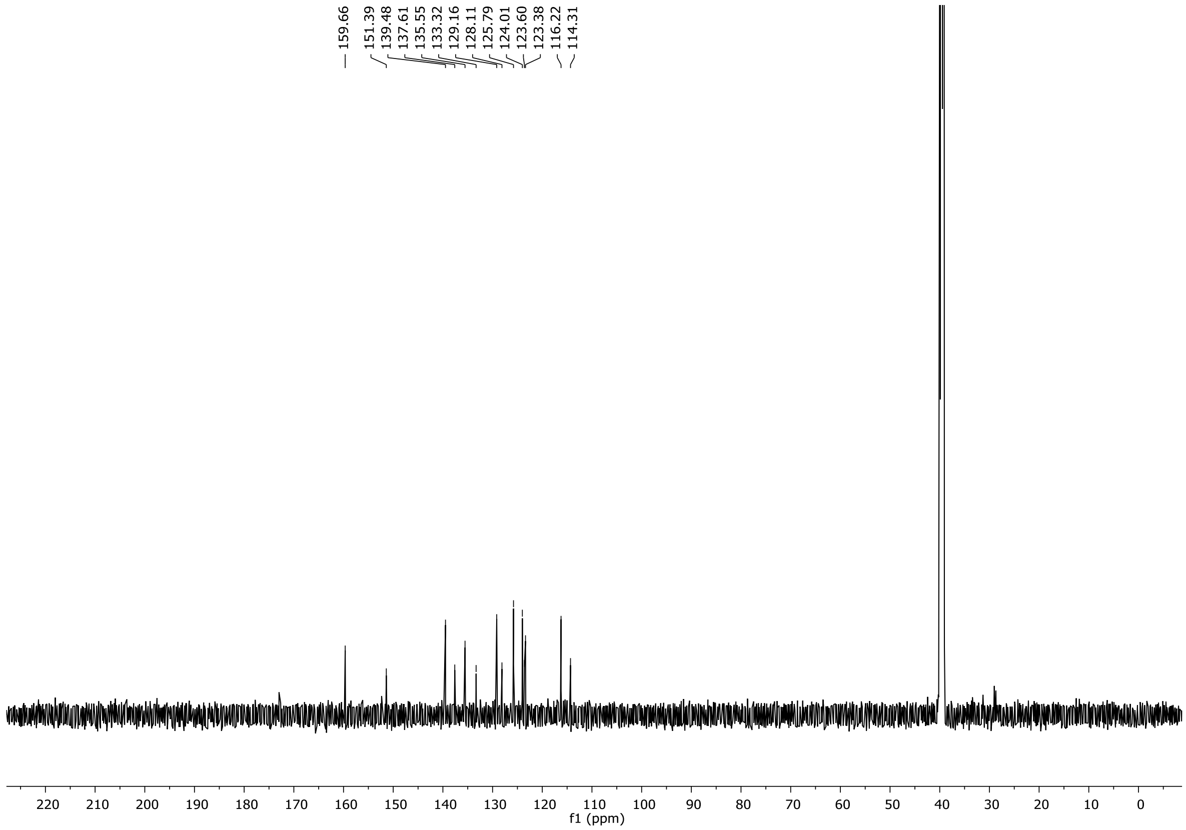

1-Hydroxy-canthin-6-one (2). COSY H-H (MeOD-d4)

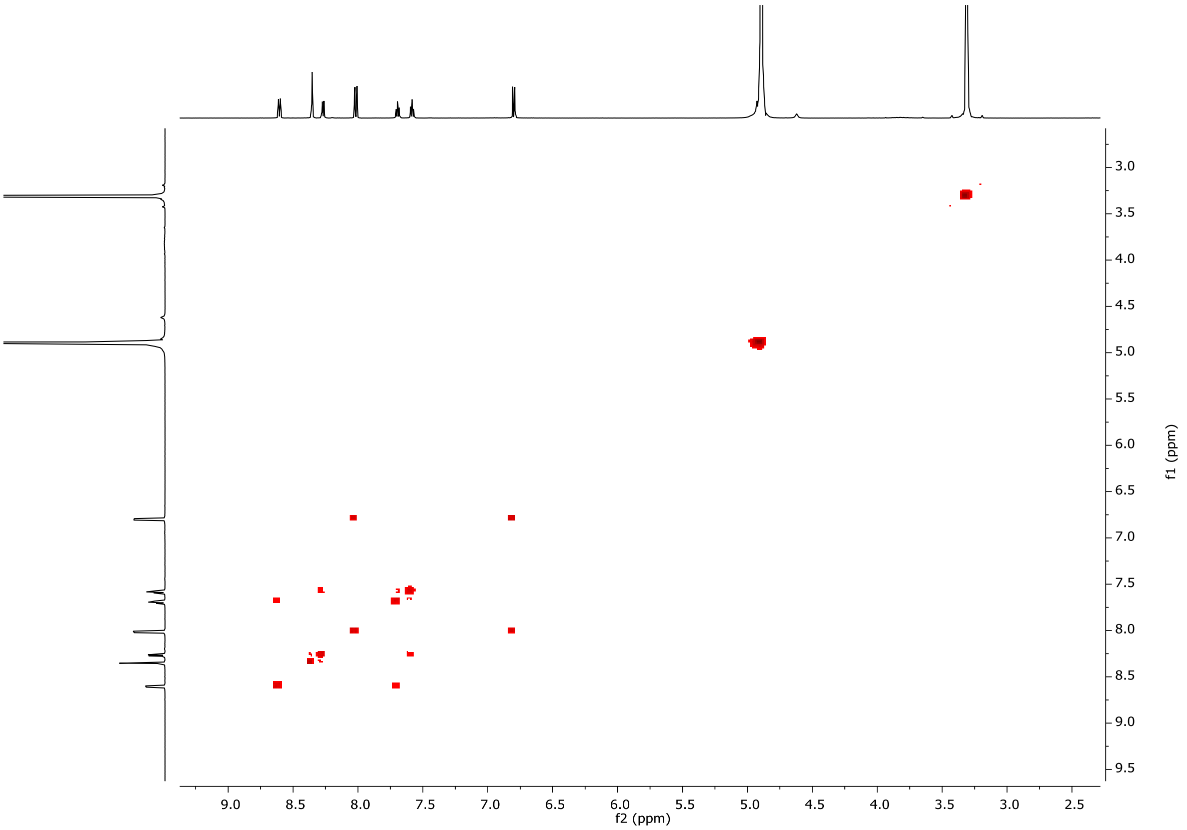

1-Hydroxy-canthin-6-one (2). HSQC (MeOD-d4)

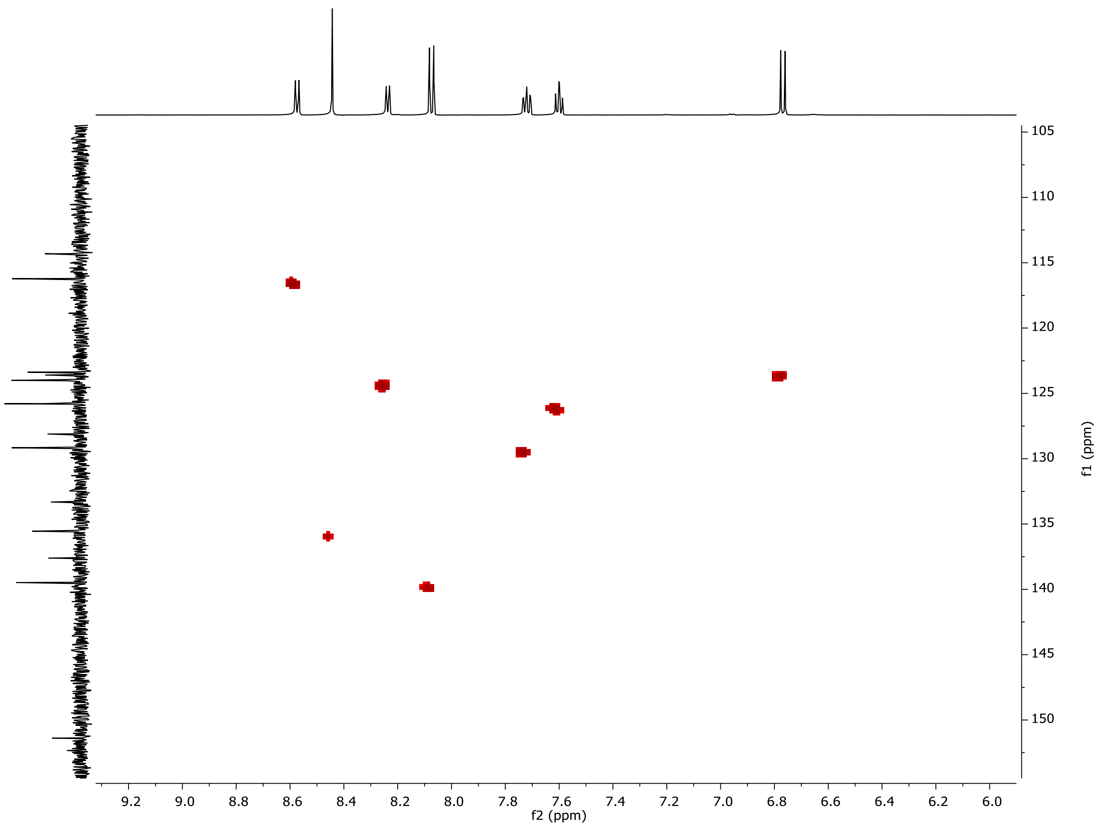

1-Hydroxy-canthin-6-one (2). HRMS.

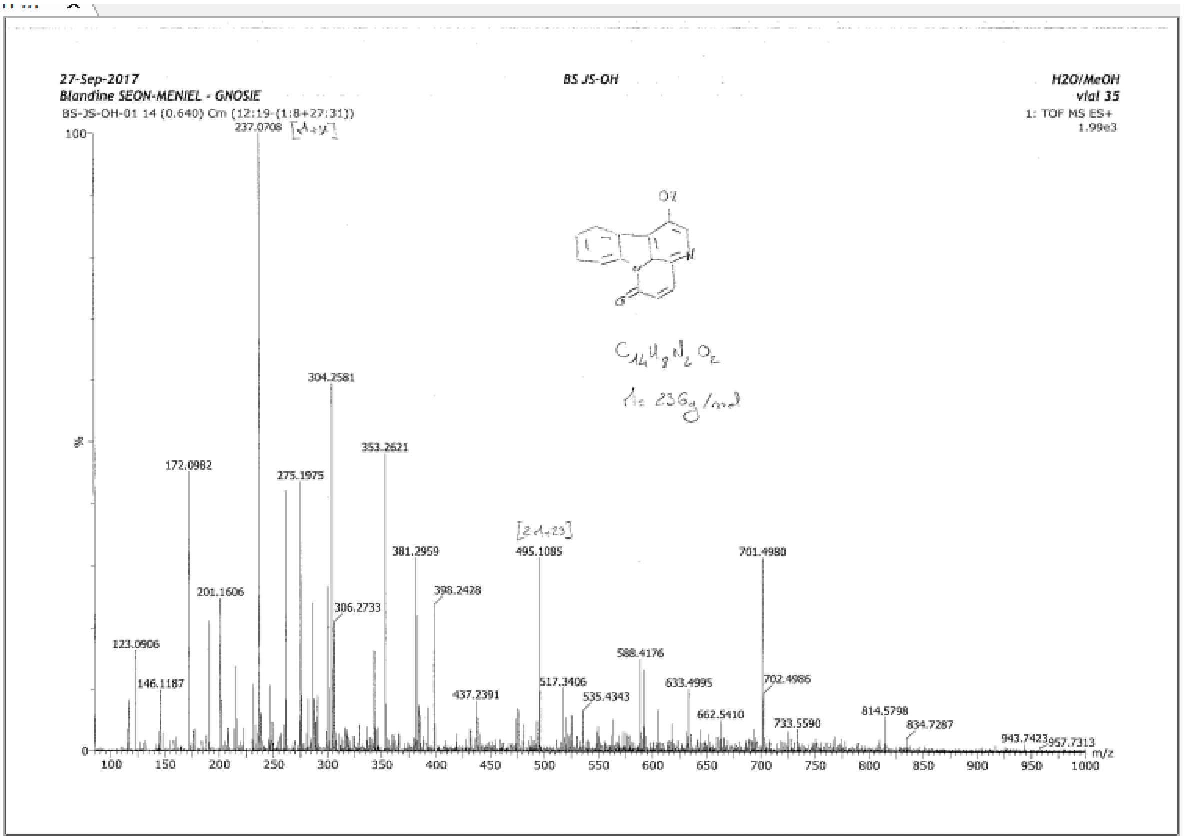

Canthin-6-one (3). ^{1}H-NMR (DMOS-d6, 600MHz).

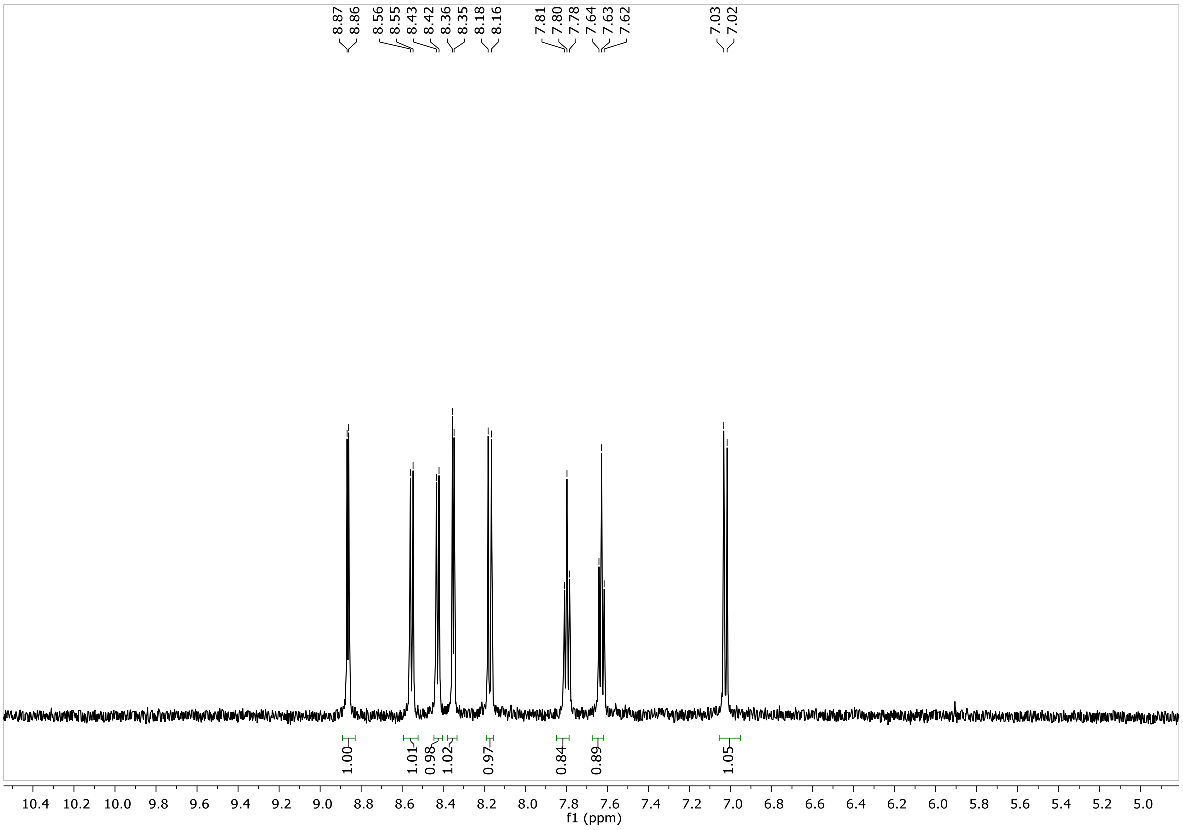

Canthin-6-one (3). HRMS

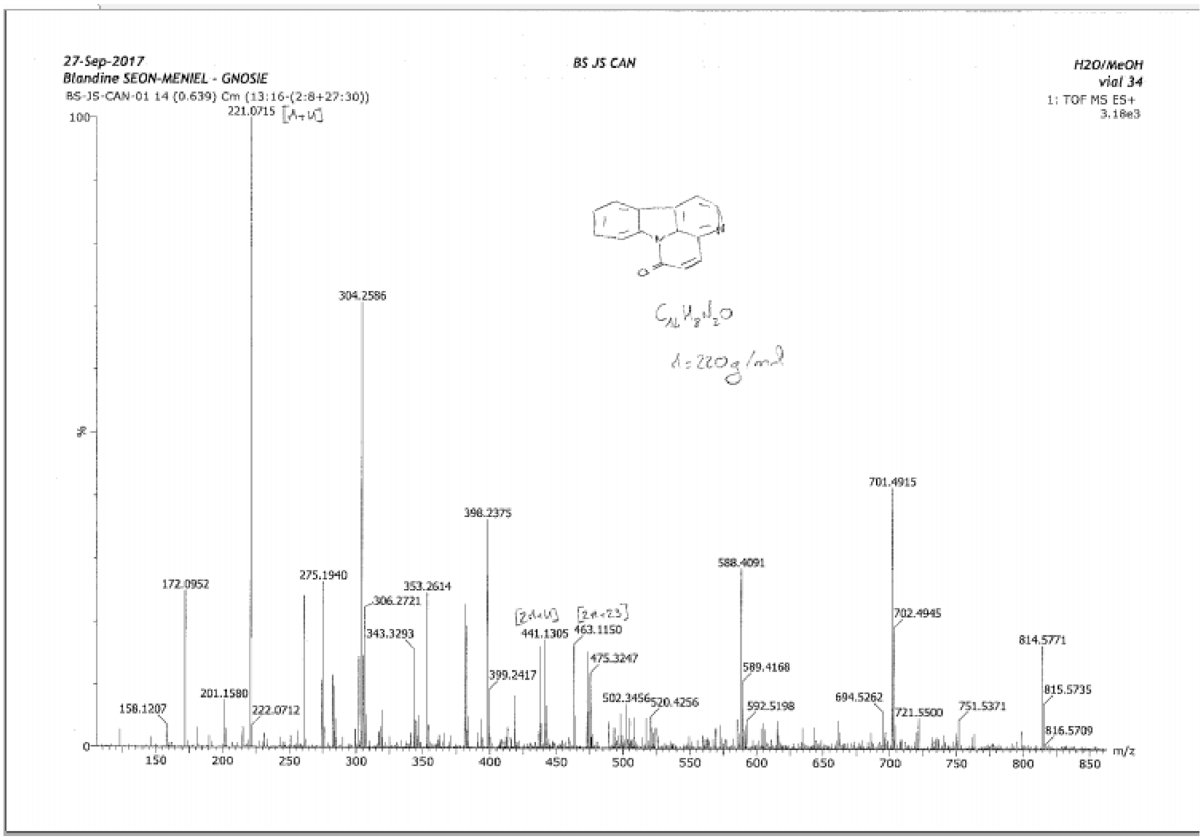

Supplement: Supplementary file 1 [file molecules-23-00934-s001.pdf]
